# Continuous sPatial‐temporal deformable image registration and 4D frame interpolation

**DOI:** 10.1002/mp.70248

**Published:** 2025-12-26

**Authors:** Xia Li, Runzhao Yang, Muheng Li, Xiangtai Li, Antony J. Lomax, Joachim M. Buhmann, Ye Zhang

**Affiliations:** ^1^ Center for Proton Therapy Paul Scherrer Institut Villigen Switzerland; ^2^ Department of Computer Science ETH Zürich Zürich Switzerland; ^3^ Department of Physics ETH Zürich Zürich Switzerland; ^4^ Department of Automation Tsinghua Univeristy Beijing China; ^5^ S‐Lab Nanyang Technological University Singapore Singapore

**Keywords:** 4D frame interpolation, continuous representation, deformable image registration, implicit neural representation, motion representation

## Abstract

**Background:**

Deformable image registration (DIR) is a crucial tool in radiotherapy for analyzing anatomical changes and motion patterns. Current DIR implementations rely on discrete volumetric motion representation, which often leads to compromised accuracy and uncertainty when handling significant anatomical changes and sliding boundaries. This limitation affects the reliability of subsequent contour propagation and dose accumulation procedures, particularly in regions with complex anatomical interfaces such as the lung‐chest wall boundary.

**Purpose:**

Given that organ motion is inherently a continuous process in both space and time, we aimed to develop a model that preserves these fundamental properties. Drawing inspiration from fluid mechanics, we propose a novel approach using implicit neural representation (INR) for continuous modeling of patient anatomical motion. This approach ensures spatial and temporal continuity while effectively unifying Eulerian and Lagrangian specifications to enable natural continuous motion modeling and frame interpolation. The integration of these specifications provides a more comprehensive understanding of anatomical deformation patterns.

**Methods:**

We propose an INR‐based approach modeling motion continuously in both space and time, named continues‐sPatial‐temporal deformable image registration (CPT‐DIR). This method fits a multilayer perception network to map the 3D coordinate (x,y,z), to its corresponding velocity vector (vx,vy,vz). Displacement vectors (▵x,▵y,▵z) are then calculated by integrating velocity vectors over time using an Euler method numerical scheme. The above spatial and temporal continuous motion design also enables continuous frame interpolation (CPT‐Interp). The DIR's and interpolation's performance were tested on the DIR‐Lab dataset and the Abdominal‐DIR‐QA dataset, using metrics of landmark accuracy (target registration error), contour conformity (Dice), and image similarity (mean absolute error).

**Results:**

CPT‐DIR clearly reduced landmark TRE from 2.79±1.88 to 0.99±1.07 mm over DIRLab and from 8.61±7.92 to 4.79±6.28 mm over the challenging Abdominal‐DIR‐QA dataset, surpassing B‐spline results across all cases. The whole‐body region MAE improved from 35.46±46.99 to 28.99±32.70 HU for DIRLab, and from 37.32±18.69 to 20.65±16.39 HU for Abdominal‐DIR‐QA. In the challenging sliding boundary region, CPT‐DIR demonstrated superior performance compared to B‐spline, reducing ribcage MAE from 75.40±86.70 HU (unregistered) to 42.04±45.60 HU and improving Dice coefficients from 89.30% to 90.56%. The training‐free CPT‐Interp method enhanced previous deep learning‐based approaches, improving upon UVI‐Net with reduced MAE (17.88±3.79 vs. 18.93±3.90) and increased peak signal‐to‐noise ratio (PSNR) (40.26±1.58 vs. 39.76±1.48), while eliminating training dataset dependencies. Both CPT‐DIR and CPT‐Interp achieved substantial computational efficiency, completing operations in under 3 s compared to several minutes required by conventional B‐spline methods.

**Conclusion:**

By leveraging the continuous representations, the CPT‐DIR method enhances registration and interpolation accuracy, automation, and speed. The method achieves high accuracy on intra‐fractional thoracic datasets and demonstrates improved performance over conventional methods in more challenging inter‐fractional abdominal registration scenarios, highlighting its potential for robust applications in radiotherapy. The improved efficiency and accuracy of CPT‐DIR make it particularly suitable for real‐time adaptive radiotherapy applications.

## 1 INTRODUCTION

In radiotherapy, accurate alignment of anatomical structures is of paramount importance for ensuring high precision treatment delivery and minimizing damage to healthy tissues.^1^ Deformable image registration (DIR) is a key technique used to achieve this alignment by estimating the dense displacement vector field (DVF) between sequential images, thereby enabling the mapping of anatomical structures from one image to another.^2^, ^3^ DIR plays a crucial role in various aspects of radiotherapy, including contour propagation, dose accumulation, and 4D dose calculation.^4^, ^5^ However, achieving robust and precise DIR in highly dynamically changed anatomical regions, such as the head‐and‐neck, remains a significant challenge.^6^ These regions are subject to complex inter‐fraction and intra‐fraction anatomical changes due to variations in patient positioning, respiration, and tissue deformation.^7^ Such changes can lead to substantial discrepancies between the planning and treatment images, compromising the accuracy of dose delivery and potentially leading to suboptimal treatment outcomes. Previous DIR methods, which rely on discrete spatial modeling with end‐to‐end mapping, often struggle to handle these complex anatomical variations.

The complexity of DIR arises from several factors, including the lack of definitive ground truth^8^ and the high degree of freedom in DVF optimization.^9^ Over the years, DIR methods have evolved from traditional approaches, such as optical flow^10^, ^11^ and elastic models,^12^, ^13^ to more advanced techniques, such as the Demons algorithm^14^, ^15^ and B‐spline registration.^16^, ^17^ These advancements have improved the performance of DIR in terms of accuracy and efficiency; however, they still have limitations, especially the slow speed^18^ and expert tuning.^19^ More recently, deep learning‐based approaches have emerged as a promising direction for advancing DIR.^20^ Methods like VoxelMorph^21^ have demonstrated the potential of unsupervised learning in improving the accuracy and speed of DIR by learning the DVF directly from image data. SynthMorph^22^ has extended these capabilities to multi‐modal image registration, enabling the alignment of images from different modalities, such as CT and MRI. Transmorph^23^ has further enhanced spatial correspondence modeling by incorporating transformer models, which can capture long‐range dependencies and improve the robustness of DIR. Despite these advancements, deep learning‐based DIR methods still face challenges, such as the requirement for large and diverse training datasets and the difficulty in generalizing to new data or imaging modalities.^24^

Beyond the DIR task, several methods have been proposed, specifically 4D medical image interpolation.^25^ SVIN^26^ employed a dual‐network strategy for capturing and interpolating volumetric motion, but its application was limited by its reliance on extensive radiation, lengthy imaging processes, and the availability of ground‐truth intermediate images for training. MPVF^27^ used multi‐pyramid voxel flows to address periodic motion in organ structures; however, it struggled with nonperiodic motions due to its discrete representation of dynamic biological processes. UVI‐Net^28^ avoids using intermediate frames through a cycle consistency model, enhancing image fidelity from limited data. Yet, its reliance on linear motion assumptions can lead to spatial distortion and inaccuracies in capturing complex physiological movements. Despite these advancements, accurately modeling anatomic changes' continuous and complex nature over time remains an ongoing challenge.

To overcome these limitations, we propose an advanced deformation modeling approach that leverages continuous spatial and temporal representations for precise registration. Our method employs implicit neural representations (INR)^29^, ^30^, ^31^ to create continuous models of DVFs that capture the fine‐grained details of anatomical deformations over time and space. By utilizing INR, we aim to move beyond conventional voxel‐based registration techniques, offering a more flexible and accurate way to model complex deformations during proton therapy. We model the registration process as a spatial‐temporal continuous flow, using a multi‐layer perceptron (MLP) network, effectively addressing sliding boundary issues and large deformations while adapting quickly to new cases without extensive hyper‐parameter tuning. The main contributions of this work are threefold. First, we introduce a novel INR‐based approach for modeling spatial‐temporal deformations in medical image registration. Second, we demonstrate the effectiveness of our method in capturing complex anatomical changes in highly dynamic regions, such as the lung. Finally, we compare our approach with state‐of‐the‐art DIR and 4D medical image interpolation methods, highlighting its advantages in terms of accuracy and robustness.

## 2 METHODS

### 2.1 Conventional approaches for DIR and interpolation

Both DIR and 4D frame interpolation rely on motion modeling, typically achieved by estimating the displacement field $\phi_{0\to 1}$, which is linear in time. Given a pair of 3D images $I_{0}$ (fixed) and $I_{1}$ (moving), where voxel $z_{0}\in Z^{3}$, a DIR algorithm estimates the DVF $\phi_{0\to 1}$ that maps the coordinates in $I_{0}$ to their corresponding locations in $I_{1}$, aligning the two images by minimizing the difference between the reference image $I_{0}$ and the warped moving image $I_{1}\circ \phi_{0\to 1}$. Since this problem is ill‐posed in general, regularization terms are introduced to constrain the solution space of $\phi_{0\to 1}$. The optimization problem for DIR can be formulated as:

(1) $$\phi_{0\to 1}^{*}=\underset{\phi_{0\to 1}}{\arg\min}D\left(I_{0},I_{1}\circ \phi_{0\to 1}\right)+\lambda R\left(\phi_{0\to 1}\right),$$

where D measures the image similarity and is usually implemented as the sum of squared differences (SSD),^32^ normalized cross correlation (NCC),^33^ or normalized gradient fields (NGF).^34^ Additionally, R with strength λ is the regularization term; possible choices for R include the classic L1 loss, total variation (TV) loss,^35^ etc. Conventional methods directly optimize the DVF map $\phi_{0\to 1}$ or use condensed representations like B‐spline,^36^ while deep learning‐based methods^14^, ^16^, ^21^, ^22^, ^23^ learn a mapping function $f:I_{0}\times I_{1}\to \phi_{0\to 1}$ from a large set of data pairs. In both paradigms, $\phi_{0\to 1}$ is modeled discretely and directionally, utilizing the volumetric or the spline‐based representation. This representation implies that $\phi_{0\to 1}:Z^{3}\to R^{3}$ maps a coordinate $z_{0}$ on the grid (where, $z_{0}\in Z^{3}$) of $I_{0}$ to a continuous location $x_{1}$ in $I_{1}$. The trilinear interpolation operator is required to calculate the displacement vector for a continuous location $x_{0}\in R^{3}$.

**FIGURE 1:**
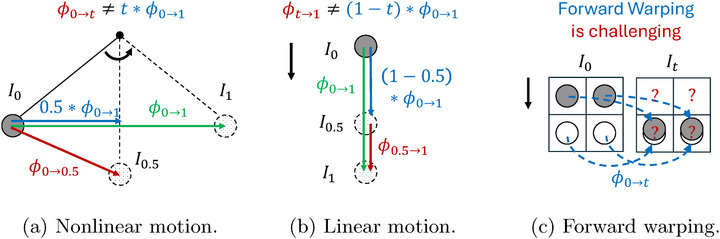
(a) In the nonlinear motion shown, $t*\phi_{0\to 1}$ does not give an accurate $\phi_{0\to t}$ . They start at the same point, but there are errors in direction and magnitude. (b) In the linear motion shown, $\left(1-t\right)*\phi_{0\to 1}$ does not give an accurate $\phi_{t\to 1}$ either. They are in the same direction, but the modeling errors are in the starting point and magnitude. (c) In the overlapping scenario shown, even with an accurate $\phi_{0\to t}$ , ambiguities arise during the forward warping of $I_{0}$: (i) What values should be assigned to the two upper pixels that are not targeted by the warping? (ii) What values should be assigned to the two lower pixels that are mapped from multiple sources?

With the estimated DVF $\phi_{0\to 1}$, we naturally have linear motion modeling. Built upon this, 4D frame interpolation estimates the intermediate frame $I_{t},t\in \left(0,1\right)$. In previous approaches, two directions have been pursued: **Forward warping**: The DVF for the intermediate frame is approximated by $\phi_{0\to t}\approx t\cdot \phi_{0\to 1}$, and a trained neural network handles the challenging task of forward warping $I_{0}•\phi_{0\to t}$. This model introduces significant errors: (a) as shown in Figure 1a, $\phi_{0\to t}\approx t\cdot \phi_{0\to 1}$ is not precise when the motion is nonlinear; (b) Forward warping suffers from holes and multiple sources mapping,^37^ as illustrated in Figure 1c. **Backward warping**: Researchers adopt the backward warping $I_{1}\circ \phi_{t\to 1}$, but this method suffers from inaccuracies in approximating $\phi_{t\to 1}$. As shown in Figure 1b, $\phi_{t\to 1}\neq \left(1-t\right)\cdot \phi_{0\to 1}$, even for the linear motion. This discrepancy arises because the vector $\left(1-t\right)\cdot \phi_{0\to 1}$ starts from a continuous location $x_{t}\in R^{3}$, while $\phi_{t\to 1}$ for warping requires the vector to start from a discrete grid‐point $z_{t}\in Z^{3}$.

### 2.2 Continuous sPtatial and temporal representation

#### 2.2.1 Spatial continuous modeling

**FIGURE 2:**
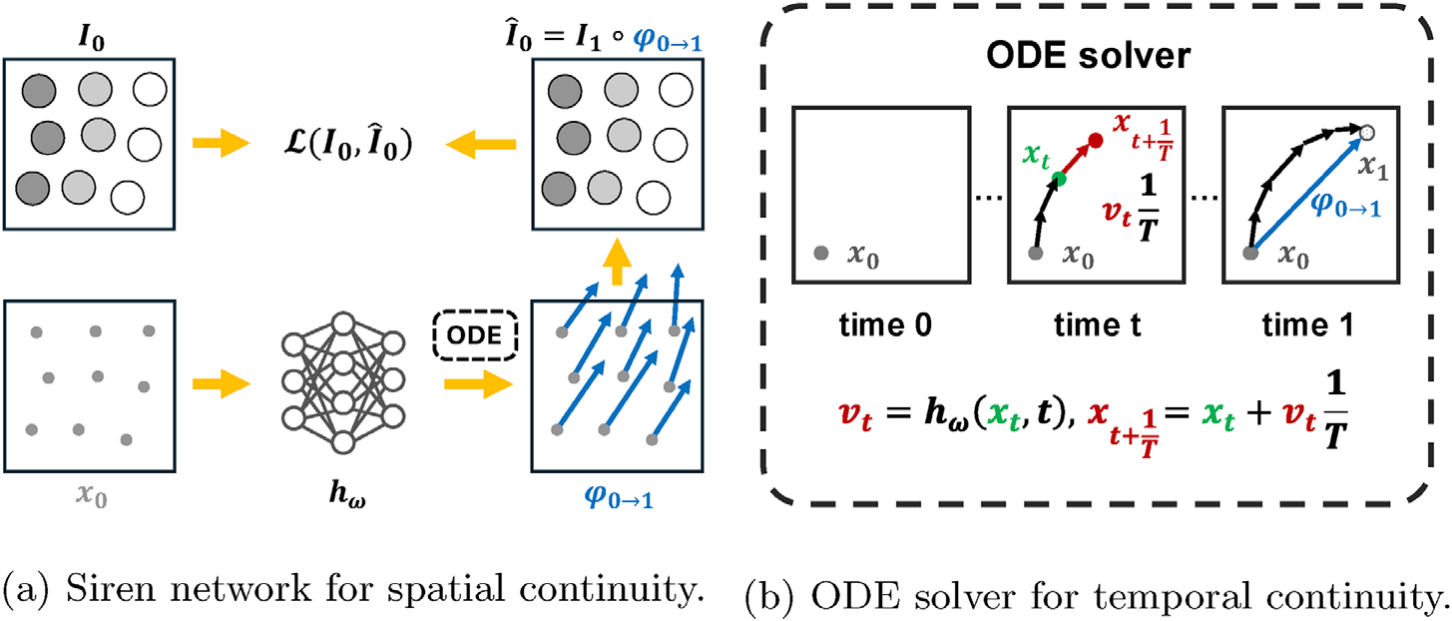
The proposed continuous sPatial and temporal (CPT) modeling. Given continuous input locations $x_{0}$, the Siren network $h_{\omega }$ estimates their corresponding velocities $\upsilon_{t}$ at time t. The full trajectory is then calculated by the ODE solver over $\upsilon_{t},t\in \left[0,1\right]$, yielding the displacement vector $\varphi_{0\to 1}$ for optimizing the DIR. The CPT motion modeling then naturally enables continuous image interpolation. CPT, continous spatial and temporal; DIR, deformable image registration; ODE, ordinary differential equation.

The above dilemma faced in DIR and video frame interpolation (VFI) estimation arises from the discrete modeling in both space and time. Only through the spatial continuous modeling, we can precisely estimate the continuous DVF $\varphi_{0\to 1}:R^{3}\to R^{3}$. To achieve this goal, we leverage the adaptivity of INR, which has achieved great success in 3D reconstruction. Instead of using INR to reconstruct the 3D image, we use it to model the displacement, resulting in $\varphi_{0\to 1}\left(x\right)=g_{\theta }\left(x\right)+x$, where $g_{\theta }$ is implemented as a Siren^30^ network, as in IDIR.^31^ The optimization target for DIR then becomes:

(2) $$\theta^{*}=\underset{\theta }{\arg\min}D\left(I_{0},I_{1}\circ \varphi_{0\to 1}\right)+\lambda_{1}R_{1}\left(\varphi_{0\to 1}\right)+\lambda_{2}R_{2}\left(\theta \right),$$

where regularization is also applied to the network parameters θ. The process is illustrated in Figure 2a, where the network is $g_{\theta }$ instead in this modeling. This approach avoids the numerical accuracy sacrifice from trilinear interpolation, as above. Additionally, as both input and output are continuous coordinates, the DVF reversing approximations become feasible. In conclusion, spatial continuity eliminates the discrete and directional limitations of conventional representations. However, achieving continuous frame interpolation remains elusive, as accurately estimating $\varphi_{t\to 1}$ is still challenging.

#### 2.2.2 Temporal continuous modeling

In ordinary differential equation (ODE)‐^38^, ^39^, ^40^ and large deformation diffeomorphic metric mapping (LDDMM)^41^‐based DIR methods, large deformations are decomposed into smaller steps to ease the burden of correspondence searching. Although we have not attempted to solve the problem of large deformations, we have found a solution for temporal continuity by decomposition. We estimate the velocity vector field (VVF) υ instead of the DVF to achieve this. The VVF can be estimated as a function of t as $\upsilon_{t}\left(x\right)=h_{\omega }\left(x,t\right)$ or independent of t as $\upsilon \left(x\right)=h_{\omega }\left(x\right)$, where ω is the parameter for the Siren network h. The DVF is then calculated from the temporal integration of the VVF:

(3) $$\varphi_{0\to 1}\left(x_{0}\right)=x_{0}+\int_{0}^{1}h_{\omega }\left(x_{t},t\right)dt,$$

where $x_{t}$ represents the particle or tissue's location at time t. For practical implementation, this needs to be estimated discretely in time, using the Euler method:

(4) $$\varphi_{0\to 1}\left(x_{0}\right)=x_{0}+\sum_{t=0}^{1}h_{\omega }\left(x_{t},t\right)\frac{1}{T},$$

where T is the maximum number of steps for the ODE solver; typically, more steps yield less loss. As illustrated in Figure 2b, this can also be recursively calculated as:

(5) $$\varphi_{0\to 1}\left(x_{0}\right)=x_{1};\;x_{t+\frac{1}{T}}=x_{t}+h_{\omega }\left(x_{t},t\right)\frac{1}{T}.$$

Since the VVF can be reversed directly, we can easily estimate the reversed DVF $\phi_{1\to 0}\left(x_{1}\right)=x_{1}-\sum_{t=1}^{0}h_{\omega }\left(x_{t},t\right)\frac{1}{T}$. Thus, the optimization target for DIR becomes:

(6) $$\begin{array}{c} \begin{array}{cc} \omega^{*} & =\underset{\omega }{\arg\min}D\left(I_{0},I_{1}\circ \varphi_{0\to 1}\right)+\lambda_{1}R_{1}\left(\varphi_{0\to 1}\right)\\ & \;+\lambda_{2}\sum_{t=0}^{1}R_{2}\left(\upsilon_{t}\right)+\lambda_{3}R_{3}\left(\omega \right)\\ & \;+D\left(I_{1},I_{0}\circ \varphi_{1\to 0}\right)+\lambda_{1}R_{1}\left(\varphi_{1\to 0}\right), \end{array} \end{array}$$

which also constrains the diffeomorphism property of the mapping.

Our proposed method distinguishes us from ODE‐based and LDDMM‐based methods, which still rely on an Eulerian specification that models only from the coordinates' perspective. These methods require discrete steps to integrate over time, involving successive warping of the VVF, such as $\phi_{0\to 1}\approx \phi_{0\to 0.5}\circ \phi_{0.5\to 1}$. The more steps involved, the more trilinear interpolation is needed, decreasing precision. In contrast, our implementation is the first to seamlessly bridge the Eulerian and Lagrangian specifications, accommodating both the coordinates' and parcels' perspectives. The first perspective allows its easy formation as a mapping network, while the second provides the possibility for parcel/tissue tracking.

### 2.3 Extension for frame interpolation

**FIGURE 3:**
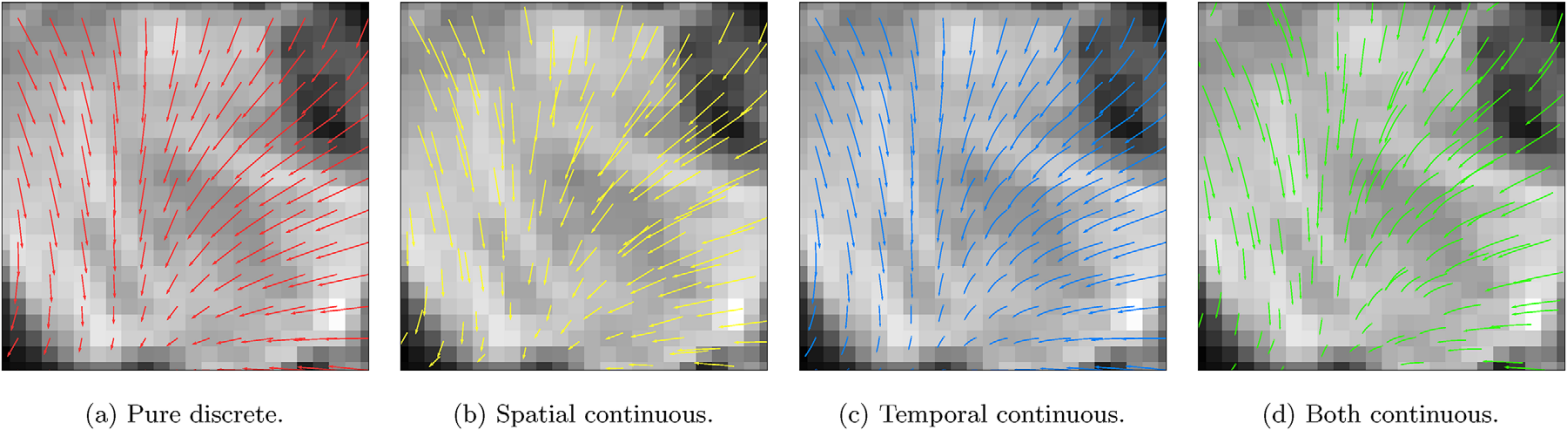
Visualization of motion under different continuity settings. (a) In pure discrete modeling, the motion arrow can only start from a discrete location on the image grid and linearly point toward the target location. (b) Temporal continuity allows for smooth trajectories, encoding the dynamic of the motion. (c) The proposed continuous spatial and temporal modeling (CPT) allows the motion to start from any continuous location while following a smooth curve. CPT, continuous spatial and temporal.

As illustrated in Figure 3d, the continuous spatial and temporal representation leads to smooth displacement curves, starting from anywhere inside the image. As a comparison, the temporal‐only continuous model allows for a smooth trajectory, but limits the starting points on the grid points $x_{0}\in R^{3}$, as illustrated in Figure 3c. Besides, spatial‐only contiguous models' trajectories can start anywhere but proceed directly as in Figure 3b. While the pure discrete can only model straight displacement vectors, shown in Figure 3a. Equipped with properties of spatial and temporal continuity, our motion model can derive the displacement vector for any time $t_{0}\in \left(0,1\right)$ to any time $t_{1}\in \left(0,1\right)$ from any location $x_{0}$, using an ODE solver (such as the Euler method:^42^)

(7) $$\varphi_{t_{0}\to t_{1}}\left(x_{t_{0}}\right)\approx x_{t_{0}}+\sum_{t=t_{0}}^{t_{1}}\upsilon_{t}\frac{1}{T},$$

where the loss of numerical precision only comes from δt.

These formulations enable us to estimate $\varphi_{t\to 0}$ and $\varphi_{t\to 1}$ more precisely than previous methods. We avoid the cumbersome forward warping because we do not utilize $\varphi_{0\to t}$ and $\varphi_{1\to t}$. Instead, we calculate frame interpolation at time t using the weighted average of $I_{1}\circ \varphi_{t\to 1}$ and $I_{0}\circ \varphi_{t\to 0}$ as:

(8) $$I_{t}=w_{1}\cdot I_{1}\circ \varphi_{t\to 1}+w_{0}\cdot I_{0}\circ \varphi_{t\to 0}.$$

The naive approach would simply **average** the weights ($w_{0}$ and $w_{1}$). However, we choose to adopt **linear** weights ($w_{1}=t,w_{0}=\left(1-t\right)$). It is worth noting that in our approach, no post‐processing or so‐called refinement network is adopted.

### 2.4 Dataset and evaluation metrics

This study utilizes the openly available DIR‐Lab dataset,^43^ tailored for research in DIR algorithm development. The dataset comprises 4DCT scans from 10 patients undergoing treatment for esophageal malignancies, with the largest displacement as 15.16±9.11 mm. Each patient dataset consists of 10 respiratory phases, spanning from 0% to 90% of the respiratory cycle in increments of 10%. The CT images have been pre‐processed to include 300 manually identified landmark points for the two extreme phases (0% and 50%), facilitating the evaluation of the landmark accuracy. Following the dataset's convention, the 0% phase (end of inhalation) CT is selected as the fixed image, while the 50% phase (end of exhalation) is chosen as the moving image. In addition to landmark points, a series of organs at risk (OARs) were extracted using Totalsegmentor^44^ and reviewed and revised by physicians to ensure adherence to clinical standards. For the interpolation task, the intermediate phases (10%–40%) are used as the ground truth to evaluate the performance.

To further assess the model's robustness and generalizability in a more challenging inter‐fractional scenario, this study also employs the recently published Abdominal‐DIR‐QA dataset.^45^ This dataset consists of 30 pairs of abdominal CT images, with each pair acquired from the same patient but on different days, presenting a difficult registration task due to significant organ deformations and inconsistent image content. The validation is facilitated by a comprehensive set of 1895 highly accurate landmark pairs identified on matching blood vessel bifurcations, averaging 63 pairs per case. Estimates of the landmark pair accuracy using digital phantoms were 0.7mm +/‐ 1.2 mm.^45^ For our evaluation, Cases 5 and 16 were excluded due to extreme field‐of‐view differences between the fixed and moving images.

### 2.5 Implementation specifications

#### 2.5.1 Specifications for deformable image registration

The CPT‐DIR algorithm was implemented into two specific models for validation and comparison: (a) the spatial continuous‐only method as end‐to‐end (denoted as E2E), employing a regularization term $R_{1}$ solely as the Jacobian determinant^31^ over the DVF; (b) the combined temporal and spatial continuous method as large deformation decomposition (denoted as LDD) utilizing the regularization term $R_{2}$ only composed of the L2 norm of the VVF to avoid Zigzag trajectory. Both models were trained with the NCC loss, and subsequently underwent two evaluation scenarios. In the first scenario, we followed the exact guidelines of the DIR‐Lab dataset to provide a fair comparison to previously submitted algorithms. The training was confined to the lung region only. Evaluation metrics in this scenario included target registration error (TRE)^46^ for landmark accuracy, mean absolute error (MAE) for image similarity in the lung region, and negative Jacobian determinant ratio of DVFs (Neg JacDet) for diffeomorphism. In the second scenario, we evaluated the DIR registration performance for the RT dose accumulation application. The training was conducted on the entire body to assess the sliding boundary region (ribcage) performance fully. No auto‐ or manual segmentation was applied in this scenario. Evaluation criteria in this scenario included the landmark accuracy TRE, and whole body MAE first. Then, the Dice (only over the DIR‐Lab dataset) and MAE were explicitly calculated for each OAR (segmented by TotalSegmentator [https://totalsegmentator.com/ ]), including lung (left and right), esophagus, vertebra, heart, spinal cord, and ribcage. As TotalSegmentator perform unsatisfactory on Abdominal‐DIR‐QA, Dice was only evaluated upon DIRLab. We systematically compared the new CPT‐DIR's performance against the performance of the classic B‐Splines algorithm (implemented in Plastimatch^47^) and the value of the corresponding metric for a scenario without registration. The significance of results was evaluated by paired *t*‐test, with statistical significance defined as p<0.05.

#### 2.5.2 Specifications for 4D frame interpolation

We implement $h_{\omega }$ as a four‐layer Siren network,^30^ with the hyper‐parameter $\omega_{0}$ set to 48 and the layer width set to 256. The output is multiplied by the size of the voxel to guarantee the initial estimation of the φ is within [‐voxel, voxel]. The T used in the ODE solver is set to the number of intermediate frames plus 1, and β used in weighted merging is set to 4. We linearly warm up the learning rate for the first 500 steps, adopt a cosine learning rate scheduler to stabilize the convergence, and train the network for 3000 steps, each with a mini‐batch of 10 000 voxels. To speed up optimization, we only used the regularization term $R_{2}$ in Equation (6) as $\sum_{t}{∥\upsilon_{t}∥}^{2}$, and neglected $R_{1}$ and $R_{3}$, with $\lambda_{2}$ set as 1e‐4. We implement CPT‐Interp with Pytorch on an NVIDIA RTX 4090 GPU and compare with previous methods for the 4D medical image interpolation, including the fully discrete conventional approach ANTs^48^ and DL‐based approach UVI‐Net,^28^ spatial continuous‐only method IDIR,^31^ temporal continuous‐only methods ORRN^49^, and NODEO.^50^ ORRN and UVI‐Net are trained over the 4DLung dataset,^51^ while all the others are case‐specific optimization methods without training. In this study, the ANTs registration framework was implemented using the TimeVaryingVelocityField transformation. Optimization was guided by minimizing a composite objective function comprising cross‐correlation (CC) and mean squared error (MSE) terms. For the IDIR method, regularization of the deformation field was achieved through the bending energy penalty. All remaining parameters for both were set to their respective default values as provided in their implementations. Except for MAE,^52^ we include peak signal‐to‐noise ratio (PSNR),^53^ NCC, structural similarity index measure (SSIM),^54^ and normalized mean squared error (NMSE) to evaluate the frame interpolation.

## 3 RESULTS

### 3.1 Results for deformable image registration

#### 3.1.1 Performance on intra‐fractional lung data

**FIGURE 4:**
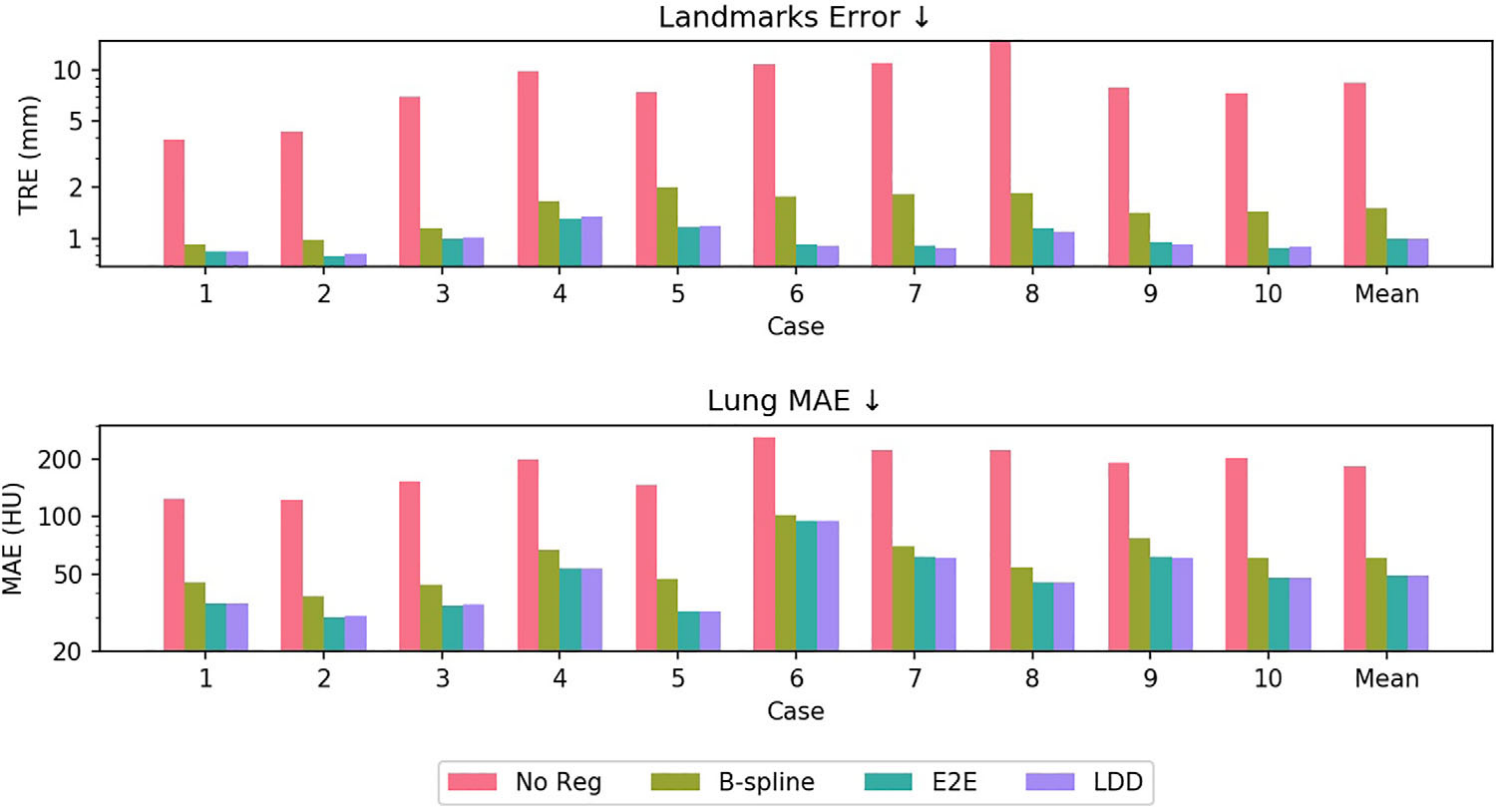
Quantitative comparisons of landmark accuracy and regional MAE between CPT‐DIRs and classic B‐spline based DIR algorithm. For fairness, the training was conducted inside the lung region. CPT, continuous spatial and temporal; DIR, deformable image registration; MAE, mean absolute error.

When training within the lung region alone, the landmark TRE for E2E and LDD models were recorded at 0.99±1.11 and 0.99±1.07 mm, respectively, greatly reducing B‐spline's TRE of 2.79±1.88 mm by more than half. By comparison, the original IDIR implementation^31^ only achieved a TRE of 1.07±1.11 mm. The leading state‐of‐the‐art conventional method, iso PTV,^35^ achieved a TRE of 0.95 mm within 3 min, whereas CPT‐DIR can achieve similar performance within only 15 s on an RTX 4090 GPU. Regarding lung MAE, both E2E and LDD models reduced B‐spline's results from 60.75±59.06 to 49.72±39.56 HU and 49.79±39.73 HU, respectively. Besides, LDD reduced the average Neg JacDet from 5e−5 (B‐spline) and 9e−5 (E2E) to 0.00. Detailed per‐case results are shown in Figure 4.

**FIGURE 5:**
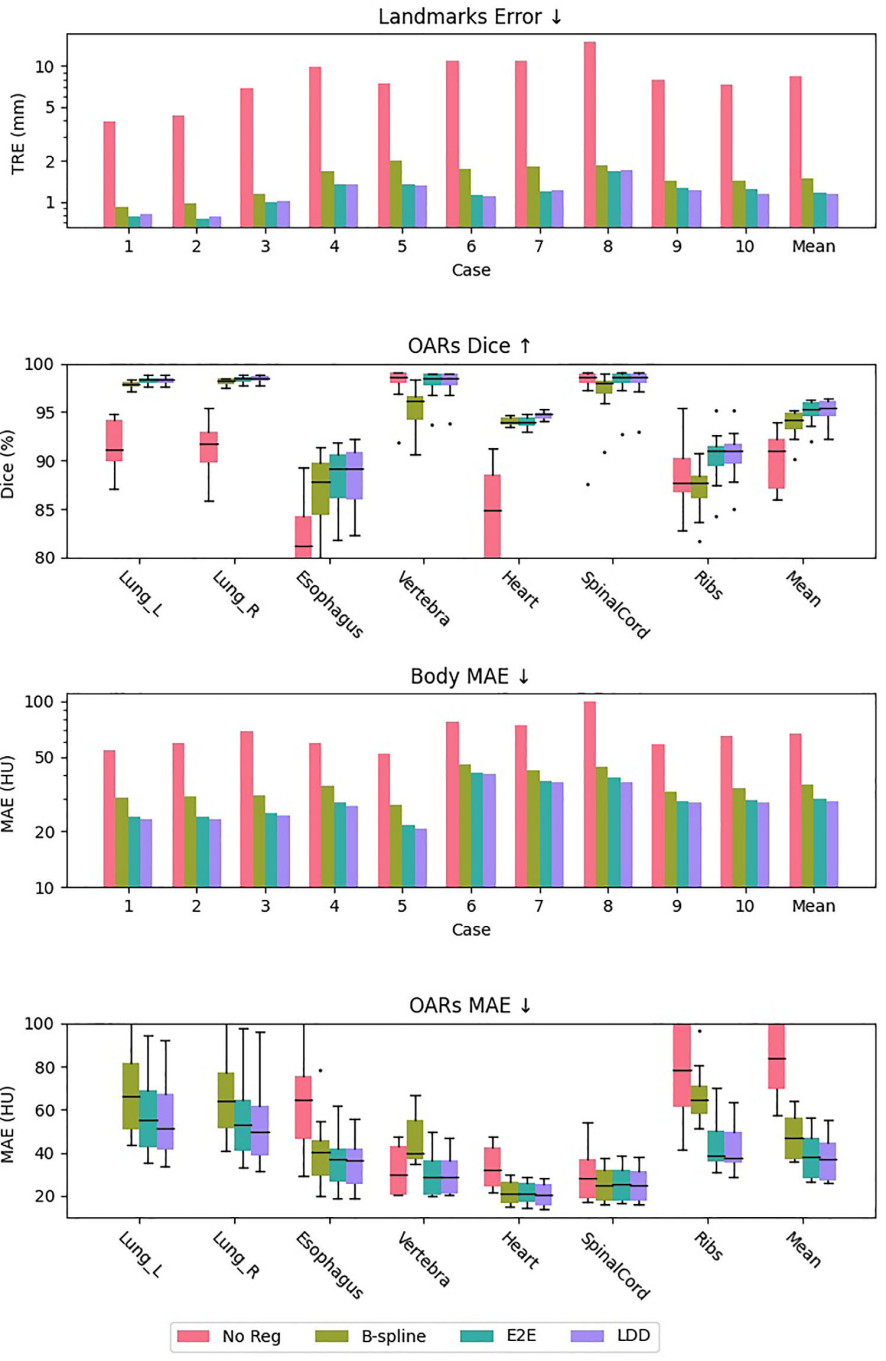
Quantitative comparison of landmark accuracy, regional MAE and OARs propagation accuracy between CPT‐DIRs and classic B‐spline based DIR algorithm. The training was conducted inside the whole‐body region. CPT, continuous spatial and temporal; DIR, deformable image registration; MAE, mean absolute error; OARs, organs at risk.

When training within the entire body, the whole‐body MAE, both E2E (29.80±34.09 HU) and LDD (28.99±32.70 HU) still considerably surpassed B‐spline (35.46±46.99 HU). The landmark accuracy degraded slightly for both the E2E and LDD models to 1.17±1.36 mm and 1.17±1.45 mm, respectively, yet remained lower than half of the B‐spline's performance. Detailed per‐case TRE, MAE, OAR‐specific Dice, and MAE are shown in Figure 5. Among all OARs, ribs are vital regions for assessing registration quality on sliding boundaries. Without registration, the MAE and Dice coefficients for ribs stood at 75.40 HU and 89.30%, respectively. B‐spline offered only marginal improvement, with metrics at 65.65 HU and 90.41%. In contrast, E2E and LDD models dramatically reduced ribcages MAE to 43.62 and 42.04 HU, respectively, while increasing Dice coefficients to 90.34% and 90.56%. Notably, while most metrics show that LDD has a slight edge over E2E, the versatility of LDD is further emphasized by its landmark error of 1.17±1.15 mm when executing a reverse trajectory.

**FIGURE 6:**
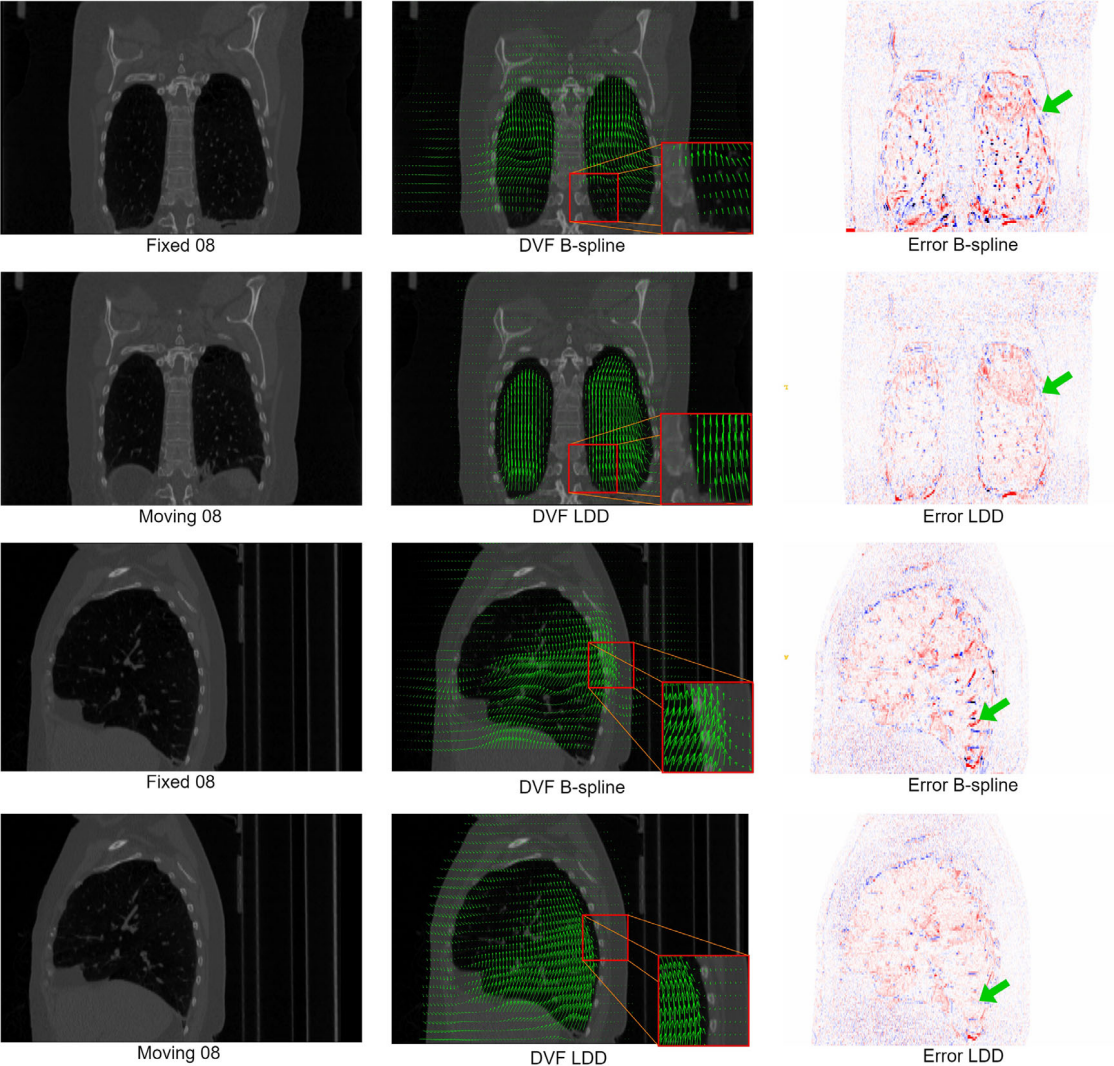
Visual comparison with B‐spline over estimated DVFs and error maps. Arrows indicate the sliding boundary region. DVF, displacement vector field.

Regarding computational efficiency, B‐spline took an average of 65.34±35.43s, while E2E and LDD processed at faster rates of 11.96±1.68 and 14.32±2.34s, respectively. Additionally, the visualizations of the resulting DVFs and error maps (between warped and fixed images) from different DIR methods can be found in Figure 6. As an example, Case‐04 (the largest landmark TRE) and Case‐08 (the largest motion) show that the new CPT‐DIR methods can reduce the difference in the ribcage region due to the improved capture of sliding boundary motion.

#### 3.1.2 Performance on inter‐fractional abdominal data

To evaluate our method under more strenuous conditions, we benchmarked it on the Abdominal‐DIR‐QA dataset, which features challenging inter‐fractional anatomical changes. As detailed in Table 1, our CPT‐DIR models demonstrated a significant advantage over the conventional B‐spline method. In terms of landmark accuracy, the LDD model achieved a mean TRE of 4.79±6.28 mm, a substantial 44% reduction compared to the B‐spline result of 8.61±7.92 mm, with $p<1\times 10^{-4}$, with also a significant improvement over E2E of 5.90±7.60 mm, $p<1\times 10^{-3}$. Critically, the LDD model excelled at maintaining a physically plausible, nonfolding transformation. This is evidenced by the Neg JacDet, where LDD achieved a near‐zero value of 0.03±0.08%. This indicates a virtually diffeomorphism‐preserving registration, marking significant improvements ($p<1\times 10^{-6}$) over both B‐spline (2.83±2.16%) and the spatial‐only E2E model (1.56±1.00%). While the relative performance gains are significant, the absolute TRE highlights the inherent difficulty of this dataset, aligning with our discussion on the challenges of inter‐fractional registration.

**TABLE 1:** Quantitative comparisons of landmark accuracy (TRE) and negative Jacobian determinant ratio (Neg JacDet) between CPT‐DIRs and classic B‐spline based DIR algorithm over the Abdominal‐DIR‐QA dataset.

Methods	No Reg	B‐spline	E2E	LDD
TRE (mm) ↓	30.80±6.55	8.61±7.92	5.90±7.60	4.79±6.28 (p<1e‐4)
MAE (HU) ↓	47.73±12.72	37.32±18.69	22.65±15.50	20.65±16.39 (p<1e‐3)
Neg JacDet (%) ↓	0.00±0.00	2.83±2.16	1.56±1.00	0.03±0.08 (p<1e‐6)

Abbreviations: CPT, continuous spatial and temporal; DIR, deformable image registration; E2E, ent‐to‐end; LDD, large deformation decomposition; MAE, mean absolute error; Neg JacDet, negative Jacobian determinant ratio; TRE, target registration error.

### 3.2 Results for frame interpolation

As shown in Table 2, CPT‐Interp outperforms all previous methods across all metrics. Noteworthy, CPT‐Interp requires no training dataset and only needs only unsupervised testing time optimization (TTO) over the test cases. This means CPT‐Interp can generalize freely to different datasets without fine‐tuning or domain adaptation. Additionally, CPT‐Interp requires no further post‐processing or refinement network. Moreover, the TTO only takes 1.96 s, and inference for all intermediate frames takes approximately 1.23s, which is much faster than the instance‐specific optimization time in UVI‐Net (70 s) and TTO in IDIR (1 min).

**TABLE 2:** Quantitative comparison of frame interpolation results.

Method	TRE ↓	MAE ↓	PSNR ↑	NCC ↑	SSIM ↑	NMSE ↓
ANTs^48^	2.61±0.90	40.50±6.72	32.97±2.36	0.541±0.102	0.895±0.028	1.849±0.539
ORRN^49^	2.40±0.75	23.51±3.50	38.30±1.07	0.691±0.085	0.950±0.012	0.576±0.258
UVI‐Net^28^	1.97±0.39	21.02±4.13	39.66±1.37	0.708±0.078	0.955±0.013	0.455±0.277
NODEO^50^	1.92±0.41	33.65±5.72	34.35±2.95	0.588±0.103	0.926±0.022	1.371±0.502
IDIR^31^	1.90±0.45	18.93±3.90	39.76±1.48	0.717±0.044	0.960±0.011	0.456±0.278
CPT‐Interp	1.87±0.39	17.88±3.79	40.26±1.58	0.731±0.045	0.964±0.011	0.414±0.271

Abbreviations: CPT‐Interp, continuous frame interpolation; MAE, mean absolute error; NMSE, normalized mean squared error; PSNR, peak signal‐to‐noise ratio; SSIM, structural similarity index measure; TRE, target registration error.

*Note*: All metrics are averaged over all intermediate frames and repeated three times.

**FIGURE 7:**
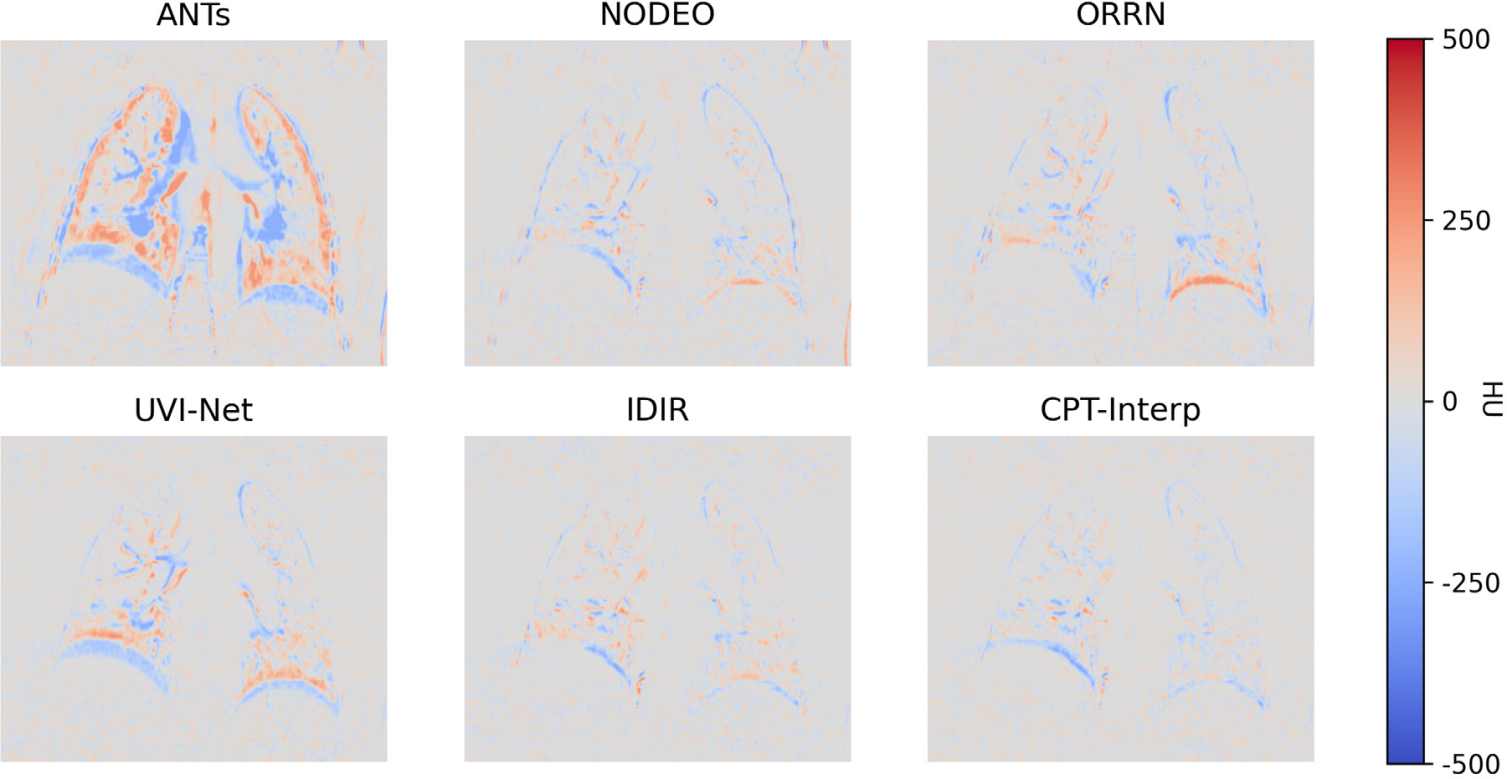
Visualization of the error maps for the interpolated frames over the DIR‐Lab dataset. The error map is calculated as the interpolated image minus the ground‐truth image. DIR, deformable image registration.

To qualitatively evaluate the performance of our CPT‐Interp method, we visualize the error maps for the interpolated frames compared to the ground truth in Figure 7. As illustrated, CPT‐Interp consistently produces error maps with fewer high‐error regions compared to the other methods, indicating a closer approximation to the ground truth.

## 4 DISCUSSION

To enhance DIR performance for RT applications, we draw inspiration from IDIR^31^ and LDDMM by incorporating spatial and temporal continuity into our LDD models. We aimed to improve intra‐fraction motion modeling closer to the nature of physical anatomy. As elaborated in the introduction, the two prevailing learning paradigms for solving DIR problems are optimization‐based and DL‐based approaches. While optimization‐based methods offer personalized modeling accuracy, they are time‐consuming due to per‐case parameter optimization. In contrast, DL‐based methods leverage large datasets for training and provide real‐time inference, but may sacrifice performance. The newly proposed CPT‐DIR and CPT‐Interp methods here are also optimized per case without individual setting parameter optimization, aligning well with the former paradigm and offering personalized enhanced accuracy. This also satisfies the nature of radiotherapy, where a large dataset is challenging to collect, clean, or align the formats. Unlike previous optimization‐based methods, our approach benefits from the computational efficiency of neural networks and GPU (benefits from DL‐based methods), reducing processing time from minutes to seconds.

The DIR problem is inherently ill‐posed and lacks a definitive ground truth, necessitating the need to parameterize the search space. Parameterization serves as a form of implicit regularization, effectively reducing the space in the solution. Voxel‐based methods address this challenge by optimizing each voxel independently, often necessitating additional regularization terms to constrain voxel relationships. Namely, DL‐based methods also operate in a voxel‐based fashion. On the other hand, B‐spline methods reduce the parameterization space from individual voxels to splines, leading to overly smooth results and difficulty in effectively handling sliding boundaries. In another direction, advanced regularizations have been proposed to solve the sliding boundary issue directly.^55^ In contrast, the new continuous motion modeling approach proposed here harnesses the power of neural networks to determine the smoothness/sharpness of each area automatically. Consequently, it can simultaneously model the smooth regions inside OARs and the sharp boundaries of the nearby organs.

However, the method's performance must be contextualized by the specific challenge at hand. As observed in our evaluation on the Abdominal‐DIR‐QA dataset, the registration task is substantially more difficult, resulting in a higher mean TRE (4.79 mm) compared to the intra‐fractional DIR‐Lab cases (0.99 mm). This inter‐fractional dataset presents challenges, such as significant organ deformation, tissue disappearance, and appearance (e.g., variations in bowel gas), which disrupt the intensity conservation assumption that DIR algorithms often rely on. While our LDD model still demonstrated a notable improvement over the conventional B‐spline method in this challenging scenario, these results indicate that further refinement may be needed to achieve clinical‐grade accuracy (<4 mm) in complex abdominal regions. Future work should focus on incorporating strategies to handle these large, non‐diffeomorphic changes more effectively. This represents a key limitation and an important direction for future research to fully validate the method's generalizability.

Another significant challenge in DIR is the management of large deformations. Conventional methods like Demons rely on local assumptions, rendering them ineffective when faced with these conditions. This issue exacerbates the search space for matching locations in other DIR methods, and significant motion may disrupt local regularization terms, affecting optimization targets. LDDMM and ODE‐based methods^49^, ^50^ address this challenge through decomposition, leveraging the typically more straightforward structure of the VVF compared to the DVF. The effectiveness of modeling large deformations through temporal continuous modeling extends beyond motion estimation, encompassing other DIR tasks in radiotherapy, such as inter‐fractional anatomic changes.

However, previous ODE‐based methods and LDDMM were limited by their reliance on voxel‐based representations, rooted in the Euler specification from the theory of fluid mechanics. Despite being designed under the Lagrange specification to track each parcel separately, their implementation was confined to the Euler specification. Our spatial continuous modeling approach estimates the VVF at any point within the image and at any time between two frames, not limited to grid points. Consequently, our implementation fully adheres to the Lagrange specification, enabling the tracking of each tissue component individually.

Another advantage of temporal continuous modeling is the ease of reversing the DVF.^56^ Conventional methods typically require double the effort to estimate bidirectional DVFs. However, in our framework, since the VVF can be directly reversed, reversing the DVF becomes straightforward. This capability enables tracking from any time between two frames, and from any location inside the image to another time and location with just one‐time training. This facilitates super‐frame rate imaging, potentially improving 4DCT reconstruction algorithms and 4D imaging pipelines in the future.

Quality assurance is crucial for commissioning DIR methods, especially for clinical applications. Landmarks provided by DIR‐Lab serve as a reliable benchmark, but their accurate labeling requires substantial human efforts. Alternatively, organ masks can be generated using open‐source segmentation tools like Totalsegmentor or other commercial tools, enabling rapid validation of fixed and moving images. In the absence of landmarks or organ masks, image similarity metrics can serve as a validation measure, albeit primarily effective in boundary regions. Moreover, robustness is another critical consideration when evaluating DIR performance. The original IDIR implementation^29^ encountered higher failure rates in some cases in DIR‐Lab, attributed to initialization schemes. We addressed this issue by adjusting initial estimations within a two‐voxel size range. Besides, the application of our proposed method in clinical pipelines requires the estimation of uncertainty, which represents the next step in our ongoing work.

## 5 CONCLUSION

In summary, this work introduces a novel approach, CPT‐DIR, that leverages continuous spatial and temporal modeling for DIR. By integrating principles of INR and LDDMM, CPT‐DIR effectively handles sliding organ boundaries and significant anatomical changes over time, overcoming the limitations of traditional voxel‐based and discrete motion modeling approaches. The tangible benefits of CPT‐DIR are evident in its high performance and efficiency on intra‐fractional thoracic data compared to classic B‐spline methods. While its robustness was further tested on challenging inter‐fractional abdominal cases, highlighting its potential, the results also underscore the need for future work to achieve consistent clinical‐grade accuracy in such complex scenarios. Furthermore, we demonstrate the extensibility of CPT‐DIR by applying it to the task of 4D medical image interpolation, referred to as CPT‐Interp. It achieves superior accuracy and speed in interpolating medical images without requiring extensive training datasets or fine‐tuning. The successful application of CPT‐DIR to both image registration and interpolation tasks highlights its potential as a unified framework for advancing medical image analysis.

## CONFLICT OF INTEREST STATEMENT

The authors declare no conflicts of interest.
